# Robust Biofilm-Forming *Bacillus* Isolates from the Dairy Environment Demonstrate an Enhanced Resistance to Cleaning-in-Place Procedures

**DOI:** 10.3390/foods8040134

**Published:** 2019-04-20

**Authors:** Ievgeniia Ostrov, Tali Paz, Moshe Shemesh

**Affiliations:** 1 Department of Food Sciences, Institute for Postharvest Technology and Food Sciences, Agricultural Research Organization (ARO), The Volcani Center, 7528809 Rishon LeZion, Israel; ievgenia.ostrov@mail.huji.ac.il (I.O.); talipaz@agri.gov.il (T.P.); 2 The Hebrew University—Hadassah, 9112001 Jerusalem, Israel

**Keywords:** dairy industry, biofilm, *Bacillus* species, biofilm derived spores, cleaning-in-place, disinfecting effect

## Abstract

One of the main strategies for maintaining the optimal hygiene level in dairy processing facilities is regular cleaning and disinfection, which is incorporated in the cleaning-in-place (CIP) regimes. However, a frail point of the CIP procedures is their variable efficiency in eliminating biofilm bacteria. In the present study, we evaluated the susceptibility of strong biofilm-forming dairy *Bacillus* isolates to industrial cleaning procedures using two differently designed model systems. According to our results, the dairy-associated *Bacillus* isolates demonstrate a higher resistance to CIP procedures, compared to the non-dairy strain of *B. subtilis*. Notably, the tested dairy isolates are highly persistent to different parameters of the CIP operations, including the turbulent flow of liquid (up to 1 log), as well as the cleaning and disinfecting effects of commercial detergents (up to 2.3 log). Moreover, our observations indicate an enhanced resistance of poly-γ-glutamic acid (PGA)-overproducing *B. subtilis*, which produces high amounts of proteinaceous extracellular matrix, to the CIP procedures (about 0.7 log, compared to the wild-type non-dairy strain of *B. subtilis*). We therefore suggest that the enhanced resistance to the CIP procedures by the dairy *Bacillus* isolates can be attributed to robust biofilm formation. In addition, this study underlines the importance of evaluating the efficiency of commercial cleaning agents in relation to strong biofilm-forming bacteria, which are relevant to industrial conditions. Consequently, we believe that the findings of this study can facilitate the assessment and refining of the industrial CIP procedures.

## 1. Introduction

Microbial contamination, caused by biofilm-forming bacteria, is one of the main threats to the quality, safety, stability and nutritional value of dairy products [1,2]. Moreover, biofilms are not only a potential source of contamination; they can also increase the corrosion rate of equipment used in the milk industry, impair heat transfer, and increase fluid frictional resistance [3]. Therefore, controlling biofilm formation is of major importance to the dairy industry [4,5,6].

Members of the *Bacillus* genus are among the most commonly found biofilm-formers in dairy farms and processing plants [7,8,9]. In addition to aggressive biofilm, these bacteria are able to form heat-resistant endospores [10,11]. To this end, the biofilm matrix can serve as an epicenter for the ripening of spores, which can be released from it and cause continuous contamination of the production environment [12,13]. Spores, as well as biofilm cells, are highly resistant to antimicrobial agents, which makes it rather difficult to eliminate them [11,14]. Moreover, biofilm matrix offers additional protection for embedded endospores, allowing their survival and colonization in the surrounding environment, when conditions are favorable [15]. In *B. subtilis*, the matrix has two main components, an exopolysaccharide (EPS) and amyloid-like fibers. Another extracellular polymer, γ-poly-dl-glutamic acid (PGA), is produced in copious amounts by some *B. subtilis* strains [16,17,18].

The main strategy to prevent biofilm formation, applied in the dairy industry, is to clean and disinfect regularly before bacteria attach firmly to surfaces [19,20]. Cleaning and disinfection in dairy processing plants have been incorporated into the cleaning-in-place (CIP) regimes, which include regular cleaning of processing equipment with alkaline and acidic liquids at high temperatures and flow velocities [4,21,22]. However, a weak point of CIP processes, evident in both industrial- and laboratory-scale systems, is their variable efficiency in eliminating established biofilms [4,21,23]. It is conceivable that biofilm formation can facilitate bacterial adaptation and survival in certain environmental niches. We therefore hypothesized that aggressive biofilm formation by dairy-associated bacteria might increase their resistance to industrial cleaning procedures.

In the present study, we evaluated the susceptibility of strong biofilm-forming dairy *Bacillus* isolates to cleaning-in-place procedures using two different model systems, which resemble industrial cleaning conditions. Our results show that the dairy-associated *Bacillus* isolates demonstrate enhanced resistance to different aspects of the CIP procedures, including mechanical, chemo-biological and disinfecting effects. Such reduced susceptibility can be attributed to robust biofilm formation by the tested dairy *Bacillus*.

## 2. Materials and Methods

### 2.1. Bacterial Strains and Growth Conditions

The following bacterial strains were used in this study: (i) dairy-associated isolates, such as *B. paralicheniformis* S127 [24,25], *B. licheniformis* MS310, *B. subtilis* MS302, *B. paralicheniformis* MS303 [24]; (ii) non-dairy isolate *B. subtilis* NCIB3610 (descendant of *B. subtilis* Marburg); (iii) poly-γ-glutamic acid (PGA)-overproducing mutant derivatives of *B. subtilis* 3610, *B. subtilis* YC295 (Δ*ywcC*) and *B. subtilis* YY54 (Δ*pgdS*) (a gift of Y. Chai [18]). *B. licheniformis* MS310, *B. subtilis* MS302 and *B. paralicheniformis* MS303 whole-genome shotgun projects are deposited at DDBJ/EMBL/GenBank, under accession numbers MIPQ00000000, MIZD00000000, MIZE00000000 respectively.

For routine growth, the strains were propagated in Lysogeny broth (LB; 10 g tryptone, 5 g yeast extract, 5 g NaCl per liter, pH 7) or on a solidified LB medium, supplemented with 1.5% agar at 37 °C.

### 2.2. Generation of Biofilm-Derived Spores

Biofilm colonies were generated at 30 °C in a biofilm-promoting medium (LBGM = LB + 1% *v*/*v* glycerol + 0.1 mM MnSO_4_) [26]. Biofilm-derived spores were obtained from colonies, as described previously [21]. Briefly, the grown (three-day-old) colonies, harvested and suspended in phosphate buffered saline (PBS; 0.01 M phosphate buffer, 0.0027 M KCl, 0.137 M NaCl per 200 mL, Sigma Aldrich, St. Louis, MO, USA), were disrupted by mild sonication (Vibra Cell, Sonics, Newtown, CT, USA; amplitude 60%, pulse 10 s, pause 10 s, duration 2 min, instrument power: 7.2 Joules per second). During sonication, the samples were kept on ice. Then, heat killing was performed at 80 °C for 20 min. Cell numbers after heat killing were quantified by the spread plating method.

### 2.3. Staining Extracellular Matrix of Biofilm-Derived Spores

Biofilm-derived spores were stained using the FilmTracer™ SYPRO^®^ Ruby Biofilm Matrix Stain (Molecular Probes, Eugene, OR, USA), according to the manufacturer’s protocol. Stained samples were visualized by confocal laser scanning microscopy (CLSM; Olympus IX81, Tokyo, Japan) at a 10 μm scale.

### 2.4. Preparation for Cleaning Tests and Enumeration of Biofilm-Derived Spores

The preparation of biofilm-derived spores for cleaning tests was performed, as described in the previous study [21]. Briefly, 200-µL aliquots of the spore suspension (containing approximately two million spores) were applied in the sampling area of stainless-steel sampling plates and dried in a biological laminar hood for 1 h. Two sampling plates were not exposed to the cleaning procedures (control). Following each cleaning test, the sampling plates were immediately subjected to abundant rinsing with tap water at RT (similar to the CIP procedures at Israeli dairy farms, where the rinsing with water stage is introduced after applying a cleaning agent). For the enumeration of the spores, the sampling area on each plate was carefully swabbed with cotton swabs, moistened in PBS buffer. Swabs from each plate were then agitated in PBS in separate test tubes. Serial dilutions from each sample were prepared, followed by spread plating on LB agar for CFU analysis. Plates were incubated for 24 h at 37 °C, before the colonies were counted. The efficiency of a cleaning procedure was evaluated by comparing the number of viable spores (attached to sampling plates), before and after cleaning.

### 2.5. Cleaning Solutions

The following cleaning solutions were used in this study: Caustic soda (NaOH), sodium hypochlorite (NaOCl) and six different commercial alkaline detergents, defined as solutions I (10–15% NaOH, 3–5% NaOCl), A (polycarboxylate, phosphates, 3.6% NaOCl), M (>5% polycarboxylate, 5–15% phosphates, 3.6% NaOCl), F (5% phosphonates, polycarboxylates), D (active chlorine, alkaline-based) and H (active chlorine, phosphates, additives, alkaline-based), which are commonly used in the Israeli dairy farms. The pH value of the tested solutions varied between 11–12; the pH of NaOH was 13; and the pH of NaOCl was 4. In accordance with the manufacturer’s recommendations, the agents were used at the following concentrations: (i) 0.5% (*v*/*v*) for solutions A, M, F, D, H; (ii) 0.6% (*v*/*v*) for solution I; (iii) 0.5% (*m*/*v*) for caustic soda and detergent H; (iv) 0.018% (*v*/*v*) for sodium hypochlorite (similar to the NaOCl concentration in working solutions of the examined cleaning agents, such as A, M and I). As a control, tap water was used (pH value around 7.7), with a standard level of hardness (50 mg/L Ca^2+^, 50 mg/L Mg^2+^), without the addition of any detergent.

### 2.6. Cleaning Test Installations

The cleaning tests were carried out either using the cleaning-in-place (CIP) model system (closely resembling the typical conditions for milking systems) [21] or using the simplified laboratory procedure, developed in this study.

#### 2.6.1. CIP Model System

The main components of the CIP model system were described in the previous study [21]. In brief, the system consists of a 5-m stainless-steel milk line (fitted with a test unit) for pumping the cleaning agents from the basin, milk releaser, and a stainless-steel return line to the basin. The test unit has T-junctions, protruding 35, 125 or 275 mm from the main loop, reflecting different degrees of cleaning difficulty. Sampling plates with the spores were mounted on the T-junctions and cleaned in the installation. The temperature of the cleaning solution during the cleaning tests was 50 °C. To generate flushing pulsation of the circulating liquid, air was introduced into the system every 8 s. The duration of each cleaning cycle was 10 min.

#### 2.6.2. Laboratory System

For cleaning tests in the laboratory system, sampling plates with the spores were placed into 100 mL plastic vessels (Yoel Naim, Rehovot, Israel), containing 50 mL of cleaning solution (preliminarily warmed to 50 °C). The samples were incubated in closed vessels at conditions simulating those in the CIP-model system (50 °C, 250 rpm) for 10 min.

### 2.7. Evaluation of the Effect of the Cleaning Agents on the Viability of Bacillus Spores

The tested solutions were added to spore suspension within tap water containing around 1 × 10^7^ CFU/mL spores. The spore suspension without the addition of detergents was used as a control. The samples were incubated in closed tubes under the conditions of the laboratory system (50 °C, 250 rpm) for 10 min. The CFU measurements of the number of viable spores were made immediately after the addition of the tested cleaning agents and following 10 min of incubation.

### 2.8. Statistical Analysis

The results of the study are the means and standard deviation (SD) of at least two independent biological experiments, performed in triplicate. The Student’s *t* test was used to calculate the significance of the difference between the mean expression of a given experimental sample and the control sample. A *p* value of <0.05 was considered significant.

## 3. Results

### 3.1. Dairy-Associated Bacillus Isolates Exhibit Robust Biofilm Phenotype Compared to B. subtilis 3610

**Figure 1:**
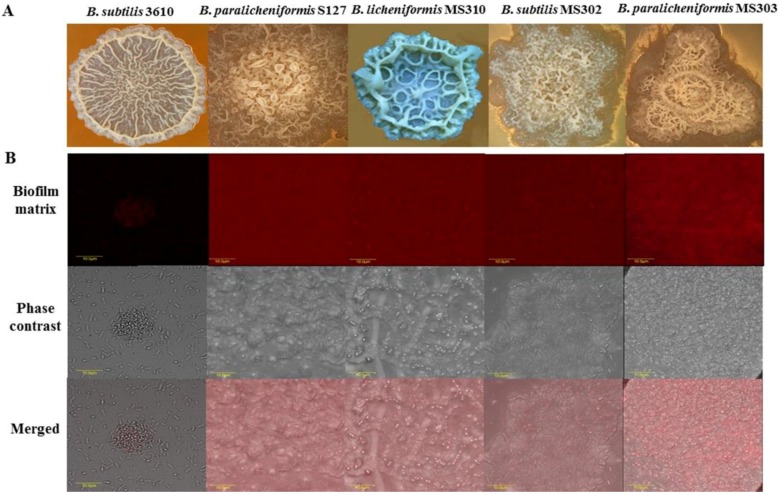
Dairy-associated *Bacillus* isolates exhibit robust biofilm formation. (**A**) Colony type biofilm formation by the tested *Bacillus* strains in the biofilm-promoting medium, LBGM. The images were taken using a stereoscopic microscope (Zeiss Stemi 2000-C; Carl Zeiss, Gottingen, Germany). (**B**) Biofilm-derived spores of the dairy *Bacillus* strains are surrounded by high amounts of the extracellular matrix. Protein components of the biofilm matrix were stained red. The samples were analyzed using a confocal laser scanning microscope (CSLM, Olympus, Japan). Scale: 10 µm.

We focused this investigation on biofilm-forming milk isolates of *Bacillus* species, which were obtained from Israeli dairy farms and recently identified and characterized [24]. The isolates were further characterized using a colony-type biofilm model for the robustness of their biofilm-forming capabilities (Figure 1; Table S1). We found notable differences in the colony-biofilm phenotype between *B. subtilis* 3610 and the dairy *Bacillus* isolates (Figure 1A). Thus, the biofilm colonies of *B. subtilis* 3610 had a complex “wrinkled” structure (shown to be a network of channels rich in biofilm matrix-producing cells [27,28]), but were not mucoid. The colonies of the tested dairy-associated strains combined an intricate "wrinkled" phenotype with the formation of highly mucoid “channel”- and “ridge”-like structures, not observed for *B. subtilis* 3610 (Figure 1A).

To support this observation, we analyzed the extracellular matrix content in the colony biofilm of the tested dairy *Bacillus* isolates and *B. subtilis* 3610 by visualizing matrix proteins. Our results indicate that biofilm cells/spores, harvested from colonies of the dairy-associated strains, could be surrounded by higher amounts of extracellular polymeric substances (EPS), compared to *B. subtilis* 3610 (Figure 1B).

### 3.2. Dairy-Associated Bacillus Isolates Display an Enhanced Resistance to the Mechanical Effect of Water Circulation

Primarily, we evaluated the susceptibility of the tested strains to water circulation in the CIP model system (closely resembling the conditions typical for milking pipes). Cleaning with water alone reflects the mechanical cleaning effect brought about by the flow of liquid in the installation [21,29]. The susceptibility of the dairy-associated *Bacillus* strains to cleaning procedures was compared to the non-dairy isolate *B. subtilis* 3610 (used as a model strain in our previous study [20]). In order to simulate dairy biofilm, we used a system that is based on the biofilm-derived spores of the tested *Bacillus*, obtained from the biofilm colonies as previously described [21].

**Figure 2:**
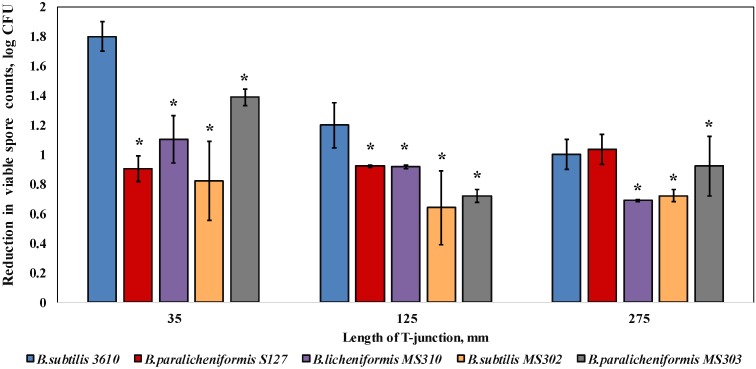
Effect of the cleaning procedure with tap water on the removal of biofilm-derived spores of the dairy-associated *Bacillus* in the CIP model system. Sampling plates, each containing approximately 2 million spores of *B. subtilis* 3610 or dairy *Bacillus* isolates, were mounted on T-junctions, protruding 35, 125, and 275 mm from the main loop of the CIP model system, and cleaned in the installation. Tap water, without the addition of any detergent, was used as the cleaning agent. A basic assumption was the similar adhesion efficiency of the spores of each tested strain in different experimental repeats (since the spores were obtained using previously validated experimental procedures [21]). The cleaning effect was evaluated by comparing the number of viable spores (attached to the sampling plates), before and after cleaning. The results represent the means and standard deviations (SD) of two independent biological experiments, performed in triplicate. * Statistically significant difference (*p* < 0.05) between the reduction in the viable spore counts of a given sample and the reduction in the spore counts for *B. subtilis* 3610 (control).

We found that the biofilm-derived spores of the dairy *Bacillus* were significantly (by 0.3–1 log) more resistant to water circulation, compared to *B. subtilis* 3610, in the case of 35 and 125 mm T-junctions (representing high levels of turbulence; Figure 2). In the samples placed into the 275-mm T-junctions (the lowest degree of turbulence available in the CIP model system), the susceptibility to cleaning was either similar (*B. paralicheniformis* S127) or lower by 0.1–0.3 log (*B. paralicheniformis* MS303, *B. licheniformis* MS310, *B. subtilis* MS302) than the control samples.

Next, we wanted to test the persistence of the examined *Bacillus* strains against the chemical effect of the commercial cleaning solutions. Since the chemical effect of the cleaning agents is less dependent on the flow turbulence, it was decided to simplify our experimental system to a lab-scale cleaning test (hereinafter referred to as the laboratory system). We first confirmed the validity of this system by comparing the strains’ ability to withstand a mechanical effect. Importantly, the dairy-associated *Bacillus* demonstrated an enhanced resistance to water circulation (by 0.6–0.7 log), compared to *B. subtilis* 3610, also during the cleaning tests performed in the laboratory system (Figure S1). A strong correlation between the results obtained in the two differently designed experimental systems indicates the reliability of the approach used.

### 3.3. Dairy-Associated Bacillus Isolates Demonstrate an Enhanced Resistance to Commercial Cleaning Agents during CIP Procedures

**Figure 3:**
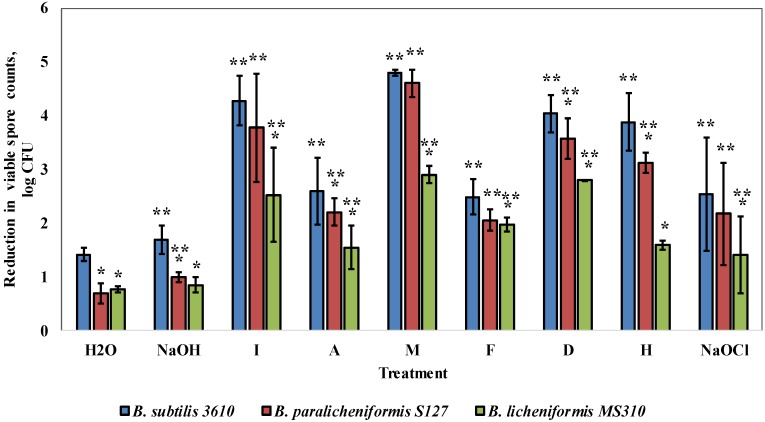
Effect of commercial cleaning agents on the removal of the biofilm-derived spores of the dairy-associated *Bacillus* in the simplified laboratory system. Sampling plates, each maintaining approximately 2 million spores of the tested *Bacillus* strains, were cleaned in the laboratory system. Caustic soda, sodium hypochlorite and the following cleaning solutions—I, A, M, F, D and H (compositions and dosages are described in Methods)—were used as the cleaning agents. The cleaning effect was evaluated by comparing the numbers of viable spores (attached to sampling plates), before and after cleaning. The results represent the means and standard deviation (SD) of two independent biological experiments, performed in triplicate. * Statistically significant difference (*p* < 0.05) between the reduction in the viable spore counts in a given sample and the reduction in the spore counts for *B. subtilis* 3610 (control). ** Statistically significant difference (*p* < 0.05) between the reduction in the viable spore counts, after treatment with a given cleaning agent, and the reduction in the spore counts for the same strain, after incubation with tap water.

Next, we evaluated the susceptibility to commercial cleaning agents of two selected dairy-associated isolates, *B. paralicheniformis* S127 and *B. licheniformis* MS310, which demonstrated the highest amount of EPS surrounding biofilm bacteria, according to a relative fluorescence analysis, in comparison to *B. subtilis* 3610 (Table S1). Consequently, we performed cleaning procedures using six different alkaline detergents, caustic soda (NaOH) and sodium hypochlorite (NaOCl) at concentrations recommended by the manufacturers. It was found that *B. licheniformis* MS310, as well as *B. paralicheniformis* S127, were more resistant to the tested solutions (up to 2.3 and 0.76 log, respectively), compared to *B. subtilis* 3610 (Figure 3). Interestingly, *B. subtilis* 3610 was particularly susceptible to agents I, M, D and H, whereas *B. paralicheniformis* S127 was highly persistent to cleaning with agent H and NaOH, but similarly susceptible to solutions I, M and F as *B. subtilis* 3610. *B. lichenifomis* MS310 was exceedingly resistant to treatment by the examined solutions, especially to agents I, M and H (Figure 3).

As indicated in the previous study [21], the biofilm removal effect of a cleaning agent includes both the mechanical effect of the liquid circulation and the chemo-biological effect from the active components, present in the agent. To gain greater insight into the mode of action of the examined solutions, we calculated their chemo-biological effect in relation to the biofilm-derived spores of the tested strains. As shown in Figure S2, *B. lichenifomis* MS310 was significantly more resistant to the chemo-biological effect of the examined solutions, compared to the other strains. At the same time, in most cases, *B. paralichenifomis* S127 was equally susceptible to the chemo-biological effect, compared to 3610. This indicates that the tested strains have varying degrees of resistance to the mechanical and chemo-biological effects of cleaning agents. Thus, the low susceptibility of MS310 to the examined solutions results from the increased resistance both to their mechanical and chemo-biological effect (Figure S3). In the case of S127, a high resistance to the majority of the tested solutions (NaOH, I, F, D) is caused mainly by the low susceptibility to the mechanical removal of spores, while the persistence to agents A and H results from a reduced sensitivity to both the mechanical and chemo-biological impacts (Figure 3; Figure S3).

### 3.4. Dairy-Associated Bacillus Isolates Demonstrate an Enhanced Resistance to the Disinfecting Effect of the Tested Agents

**Figure 4:**
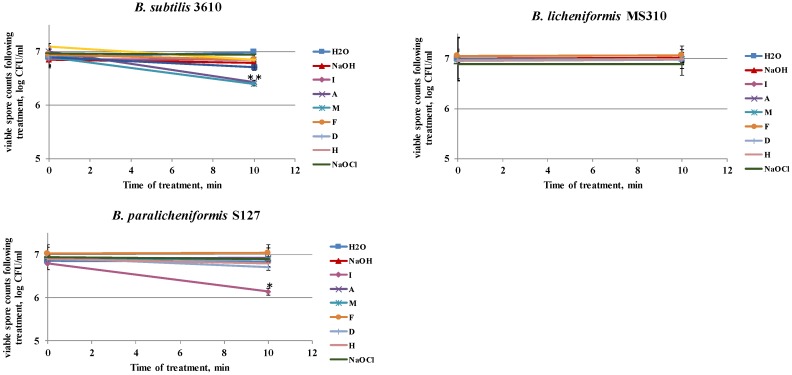
Effect of the examined cleaning agents on the viability of the biofilm-derived spores of the tested *Bacillus* strains. Caustic soda, sodium hypochlorite, and different cleaning solutions—I, A, M, F, D, and H (compositions are described in Methods)—were added to the tubes, with spore suspension of the tested *Bacillus* isolates. Spore suspension, without any detergent, was used as the control. The effect on spore viability was evaluated by comparing the numbers of viable spores in the control and after the treatment with the tested agents (following 10 min of incubation at 50 °C, 250 rpm). The results represent the means and standard deviation (SD) of two independent biological experiments, performed in duplicate. * Statistically significant difference (*p* < 0.05) between the viable spore counts in a given sample versus the spore counts after cleaning with water (control).

Primarily, we determined the ability of the tested agents to remove surface-attached spores, without affecting the viability (cleaning effect) and/or inactivating the spores (disinfecting effect). For this, spore suspensions were incubated with each of the tested agents under the conditions of the laboratory system. We found that the examined agents had different influences on the viability of the biofilm-derived spores of the tested strains (Figure 4). Thus, solutions D and M notably reduced the spore counts of *B. subtilis* 3610, after 10 min of incubation (Figure 4); there was a 0.5 log reduction in the viable spores for S127, after incubation with solution I; while none of the tested solutions affected the viability of the MS310 spores. Interestingly, NaOCl, commonly used as a disinfecting agent, did not influence the viability of the tested strains at the examined concentration (the dosage widely used in industrial cleaning agents; Figure 4).

**Figure 5:**
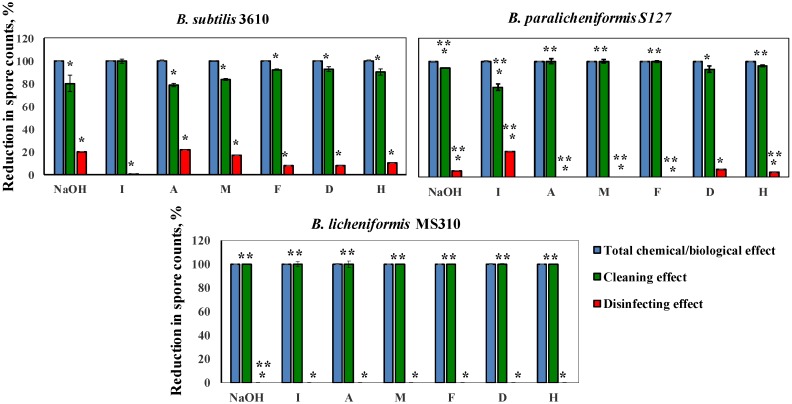
Correlation between the cleaning and disinfecting effects of the examined agents for each tested strain. Caustic soda and different cleaning solutions—I, A, M, F, D, and H (compositions are described in Methods)—were added to the tubes, with spore suspension, of the tested *Bacillus* isolates and incubated for 10 min at 50 °C, 250 rpm. The ability of a cleaning agent to reduce the number of viable spores was defined as the disinfecting effect. The percentage of the disinfecting effect was compared to the total chemical/biological effect of a cleaning agent (taken as 100%). The difference between the total chemical/biological effect of a cleaning agent and the disinfecting effect was defined as the cleaning effect. The results represent the means and standard deviation (SD) of two independent biological experiments, performed in duplicate. * Statistically significant difference (*p* < 0.05) between the reduction in the spore counts due to the cleaning or disinfecting effects versus the total chemo-biological effect of a tested agent. ** Statistically significant difference (*p* < 0.05) between the reduction in the viable spore counts in a given sample and the reduction in the spore counts for *B. subtilis* 3610 (control).

Next, we determined a correlation between the cleaning and disinfecting effects of the tested detergents. Thus, we defined the ability of a cleaning agent to reduce the number of viable spores after 10 min of a cleaning cycle, as a disinfecting effect. We compared the percentage of the disinfecting effect to the total chemo-biological effect of a cleaning agent (taken as 100%). The difference between the total chemo-biological effect of the tested agent and the disinfecting effect was defined as the cleaning effect [21]. As can be inferred from Figure 5, the ratio between the cleaning and disinfecting effects of the examined detergents differed for the tested strains. Thus, the removal of the MS310 spores was due solely to the cleaning effect of the tested solutions. *B. paralicheniformis* S127 was significantly more resistant to the disinfecting effect of agents A, M, F, H, and NaOH, compared to *B. subtilis* 3610, but much more susceptible to the disinfecting effect of solution I (Figure 5). Overall, the chemo-biological effect of the tested agents was mostly due to the removal of surface-attached spores (cleaning effect) and not to disinfecting.

## 4. Discussion

It becomes increasingly clear that biofilm formation by *Bacillus* species can facilitate their survival in the dairy environment [11,21]. Our current study investigated the effect of CIP procedures on strong biofilm-forming dairy *Bacillus*, compared to the non-dairy *B. subtilis* 3610, using differently designed model systems. As in our previous study [21], we used biofilm-derived spores to simulate the type of hygiene problem common in practice. Thus, similarly to actual dairy biofilm, biofilm-derived spores combine the presence of biofilm matrix [21] and a high content of spores [29,30]. Moreover, the resistance of vegetative cells/spores to cleaning and disinfection can be greatly enhanced by the presence of EPS [21,31]. At the same time, the presence of spores within the *Bacillus* biofilm may also modify biofilm properties, e.g., interaction forces [12].

In the current study, two model systems were used to ensure that the enhanced resistance of the dairy isolates to cleaning procedures is observed under different experimental conditions, which are relevant to the industrial CIP systems. Moreover, the design of the CIP system, employed in our previous study does not allow for the evaluation of the disinfecting effect of the cleaning agents on *Bacillus* spores directly in this system [21]. The laboratory system, developed in this study, provides sufficient conditions both for determining the mechanical, chemo-biological and disinfecting effects of the cleaning agents.

A first notable finding of the study was the enhanced resistance of the dairy *Bacillus* to the mechanical effect of liquid circulation. Thus, the most expressed difference in cleaning susceptibility between the dairy-associated strains and *B. subtilis* 3610 was observed at high levels of turbulence (35- and 125-mm T-junctions, CIP model system; Figure 2). In the case of a lower turbulence (275-mm T-junction), the difference between the dairy *Bacillus* isolates and the non-dairy strain is markedly decreased, and for some strains, it was insignificant (Figure 2). These results suggest that the protective effect of *Bacillus* biofilm matrix is most strongly expressed under a high turbulence of liquid flow. Previous studies demonstrate that a high turbulence may facilitate the removal of surface-attached bacteria [21,32,33,34], but may also increase the rate of attachment by bringing the microbial cells and the substrate in close proximity [35]. Thus, biofilm formation by the dairy-associated *Bacillus* can be detrimental not only in so-called “dead legs” (equipment details, in which the flow of liquid is significantly less turbulent), but also in main pipelines.

Furthermore, we showed that the biofilm-derived spores of the dairy *Bacillus* isolates are much more resistant to commercial cleaning agents, compared to *B. subtilis* 3610. Presumably, the causes of this resistance differ between the tested strains. Thus, the biofilm-derived spores of MS310 are, apparently, less susceptible both to the mechanical and chemo-biological effects of the employed solutions (Figures S2 and S3). At the same time, *B. paralicheniformis* S127 has the highest resistance to the mechanical removal of spores but shows a variable susceptibility to the chemo-biological effect of the tested agents.

As shown in our previous study [21], the chemo-biological effect of cleaning agents comprises a disinfecting effect (inactivating bacteria) and/or removal of them from the surfaces of dairy equipment (cleaning effect). According to our results, the dairy *Bacillus* isolates are significantly less susceptible to the disinfecting effect of the tested agents, compared to the non-dairy strain (except solution I in the case of S127; Figure 4; Figure 5). The observed differences in the mechanical and chemo-biological effects between the tested strains might be explained by the dissimilarities in the biofilm structure. For instance, a correlation between colony biofilm phenotype of the tested strains, and their resistance to the cleaning procedures, was observed (Figure 1). Thus, the dairy-associated *Bacillus*, characterized by a mucoid biofilm phenotype, were less susceptible to mechanical and chemo-biological effects during the CIP procedures. Since biofilm matrix components can be responsible for binding and/or neutralizing detergents and antimicrobial agents [36,37], differences in the matrix structure/composition can lead to differences in cleaning and/or disinfection susceptibility. Thereby, the biofilm matrix composition was shown to affect the susceptibility of food-associated staphylococci to cleaning and disinfection agents, with polysaccharide matrix-producing strains being more resistant to the lethal effect of benzalkonium chloride [38]. Likewise, the efficiency of monochloramine disinfection was dependent on the quantity and composition of EPS in *Pseudomonas* biofilms. Protein-based EPS-producing *P. putida* was less sensitive to monochloramine than polysaccharide-based EPS-producing *P. aeruginosa*, since monochloramine had a selective reactivity with proteins over polysaccharides [39]. According to Bridier et al. (2011) [40], the biofilm of the *P. aeruginosa* clinical isolate, in which a high delay of benzalkonium chloride penetration is recorded, was characterized by a large quantity of proteinacious matrix. Moreover, the authors report that, in *P. aeruginosa*, resistance to antimicrobial agents is intimately related to the inherent three-dimensional organization of cells into the exopolymeric matrix. Therefore, the low sensitivity of the dairy *Bacillus* isolates to the CIP procedures (compared to *B. subtilis* 3610) may be connected to differences in the structure/composition of the biofilm matrix.

**Figure 6:**
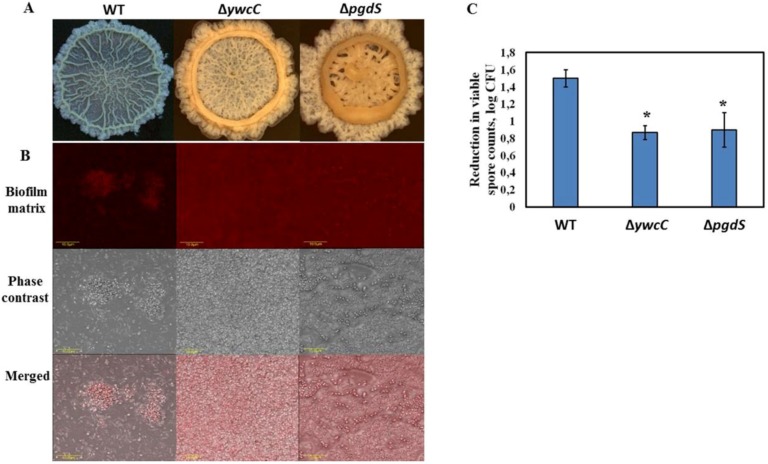
PGA-overproducing derivatives of *B. subtilis* 3610 exhibit increased resistance to the CIP procedures due to enhanced biofilm formation. (**A**) Colony biofilm formation by the tested *Bacillus* strains in the biofilm-promoting medium, LBGM. The images were taken using a stereoscopic microscope (Zeiss Stemi 2000-C; Carl Zeiss, Gottingen, Germany). (**B**) Biofilm-derived spores of the PGA-overproducing *B. subtilis* strains are surrounded by high amounts of extracellular matrix. Protein components of the biofilm matrix were stained red. The samples were analyzed using a confocal laser scanning microscope (CSLM, Olympus, Japan). Scale: 10 µm. (**C**) The effect of water circulation on the removal of biofilm-derived spores of the PGA-overproducing derivatives of *B. subtilis* 3610 in the laboratory CIP system. * Statistically significant difference (*p* < 0.05) between the reduction in the viable spore counts in a given sample and the reduction in the spore counts for *B. subtilis* 3610 (control).

Importantly, mucoid colony formation, observed for the dairy *Bacillus* isolates, was viewed as a hallmark of poly-γ-glutamic acid (PGA) production in multiple previous studies [17,18]. Significant production of PGA could result in a stronger attachment to surfaces due to its adhesive properties [41]. To this end, PGA-overproducing derivatives of *B. subtilis* 3610 (*B. subtilis* YC295 and *B. subtilis* YY54) were significantly more resistant to the mechanical effect of water circulation, compared to the wild type (Figure 6C). Notably, biofilm colonies of these mutant strains were more mucoid, compared to the WT (Figure 6A). Moreover, the biofilm-derived spores of PGA-overproducing *B. subtilis* were surrounded by higher amounts of proteinaceous extracellular matrix, which resembles the tested dairy *Bacillus* isolates (Figure 6B). Therefore, the presence of PGA in the biofilm matrix of the examined bacterial strains may be one of the factors enhancing resistance to the CIP procedures. We believe that the role of PGA and other presumptive EPS components of the dairy-associated *Bacillus* in relation to cleaning and disinfecting agents is an important subject for further investigation.

Relatively low cleaning and, especially, disinfecting effects of the tested solutions (Figure 5) might lead to undesirable implications regarding the hygiene level in dairy environments. For instance, the rapid recovery of biofilms after inappropriate disinfectant treatment is often observed. This may be due to the re-growth of surviving cells, residual biofilm, providing a conditioning layer for further cell attachment, or the selection of resistant microorganisms that survive and thrive after antimicrobial treatment [5]. In addition, biofilm cells exposure to low (sub-lethal) concentrations of disinfecting compounds, including chlorine-based detergents, can stimulate further biofilm development [10,42,43]. Therefore, we speculate that the composition of commercial CIP agents should be revised and evaluated under the experimental conditions suggested in this study.

## 5. Conclusions

We demonstrated in this study that the dairy-associated *Bacillus* isolates are characterized by an enhanced resistance to different aspects of the CIP procedures, such as the mechanical, chemo-biological, and disinfecting effects, compared to the non-dairy *Bacillus.* Such increased resistance can be attributed to robust biofilm formation by the tested dairy *Bacillus.* The results of the study underline the importance of revising the composition of commercial cleaning agents and evaluating their efficiency in relation to strong biofilm-forming bacteria, relevant to industrial conditions. To this end, the biofilm-derived spores of the dairy-associated *Bacillus*, examined in this study, can be used as an appropriate model for assessing and refining the CIP procedures.

## Supplementary Materials

The following are available online at https://www.mdpi.com/2304-8158/8/4/134/s1, Figure S1: Effect of the cleaning procedure with tap water on removal of the biofilm-derived spores of the dairy-associated *Bacillus* in the simplified laboratory system, Figure S2: Chemo-biological effect of the commercial cleaning agents on removal of the biofilm derived spores in the laboratory CIP system, Figure S3: Correlation between mechanical and chemo-biological effect of the examined agents in relation to the removal of the biofilm derived spores in the laboratory CIP system, Table S1: Relative quantity of the matrix, surrounding biofilm-derived spores of the dairy-associated *Bacillus* isolates and *B. subtilis* 3610.
