# Supplementary material for: Robust Biofilm-Forming Bacillus Isolates from the Dairy Environment Demonstrate an Enhanced Resistance to Cleaning-in-Place Procedures
Source: Foods. 2019 Apr 20;8(4):134. doi: 10.3390/foods8040134 (PMC6518050; doi:10.3390/foods8040134)
Supplement: Supplementary file 1 [file foods-08-00134-s001.pdf]

Table S1. Relative quantity of the matrix, surrounding biofilm-derived spores of the dairy-associated *Bacillus* isolates and *B. subtilis* 3610.

Strain	Relative fluorescence intensity (proteinaceous components of biofilm matrix)
B. subtilis 3610	8 ± 2
B. paralicheniformis S127	100 ± 9
B. licheniformis MS310	89 ± 9
B. subtilis MS302	73 ± 5
B. paralicheniformis MS303	80 ± 13

The relative fluorescence intensity values are based on the data obtained using CLSM. The Image J program was used to analyze the relative quantities of extracellular matrix surrounding the biofilm cells/spores.

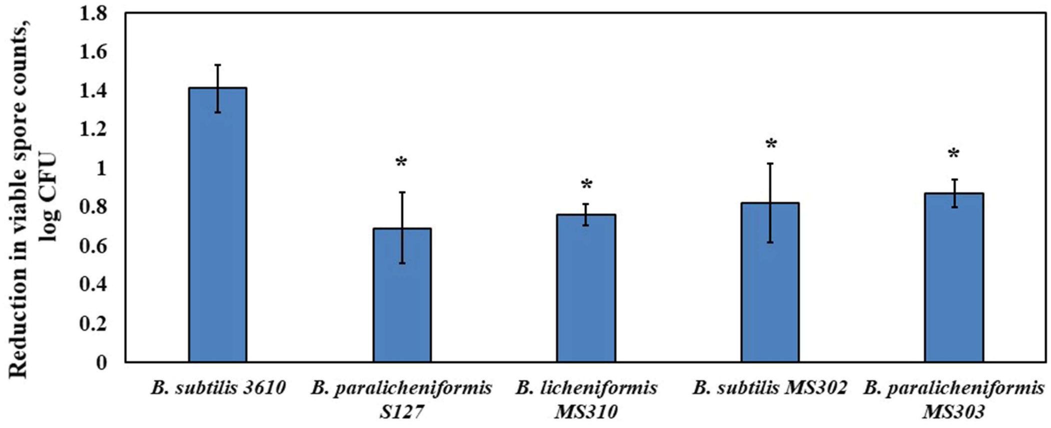

Figure S1. Effect of the cleaning procedure with tap water on removal of the biofilm-derived spores of the dairy-associated *Bacillus* in the simplified laboratory system. * – statistically significant difference ($p < 0.05$) between the reduction in the viable spore counts in a given sample and the reduction in the spore counts for *B. subtilis* 3610 (control).

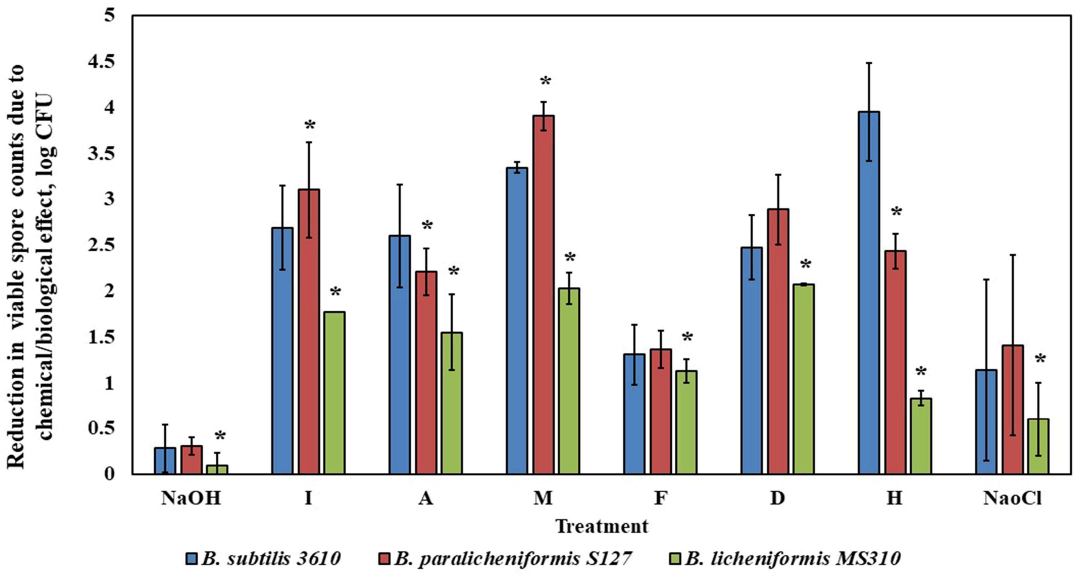

Figure S2. Chemo-biological effect of the commercial cleaning agents on removal of the biofilm derived spores in the laboratory CIP system. * – Statistically significant difference ($P < 0.05$) between

the reduction in the viable spore counts in a given sample and the reduction in the spore counts for *B. subtilis* 3610 (control).

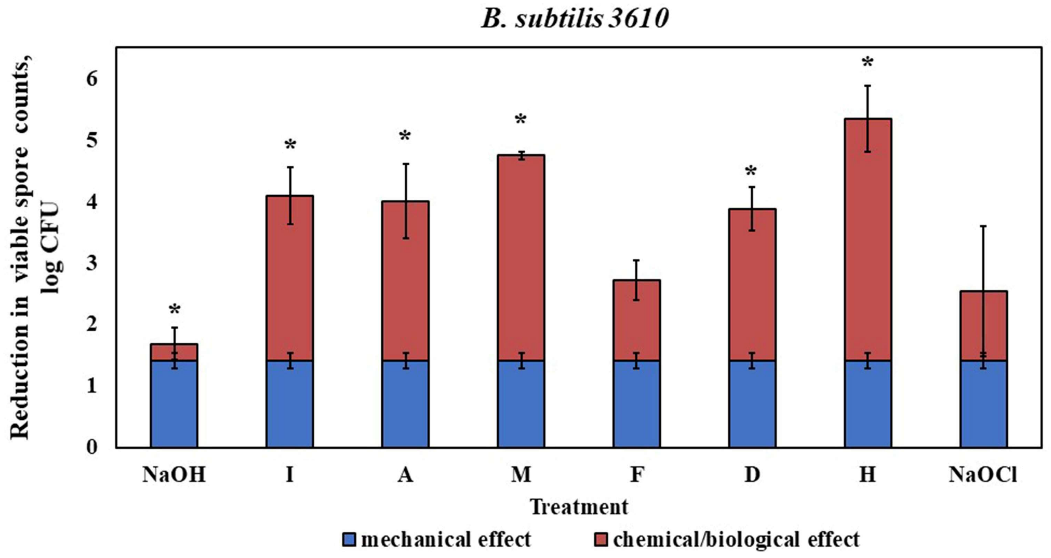

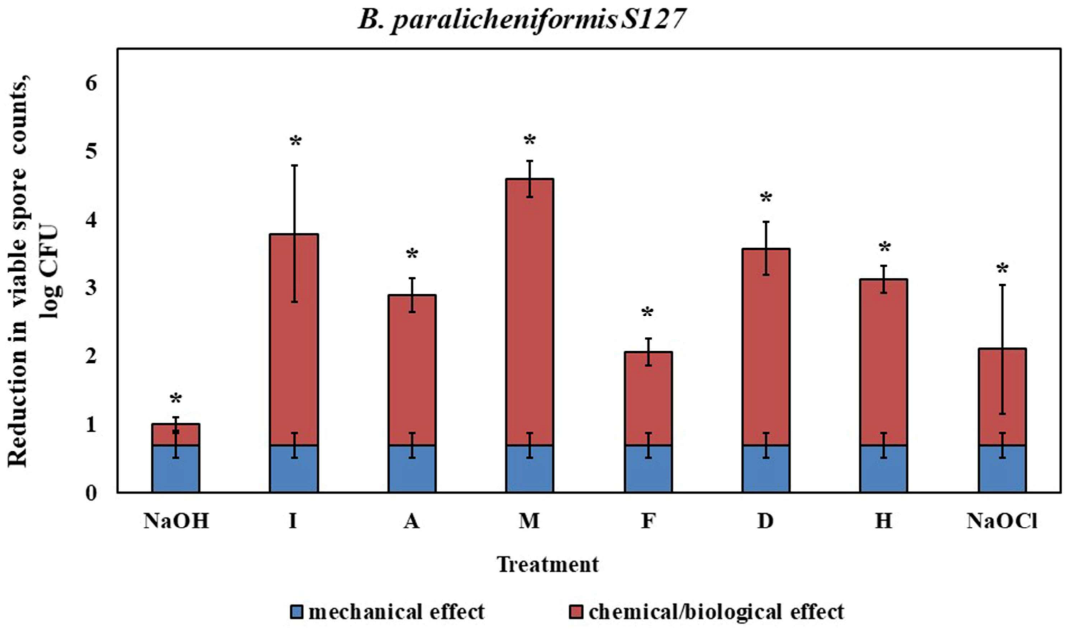

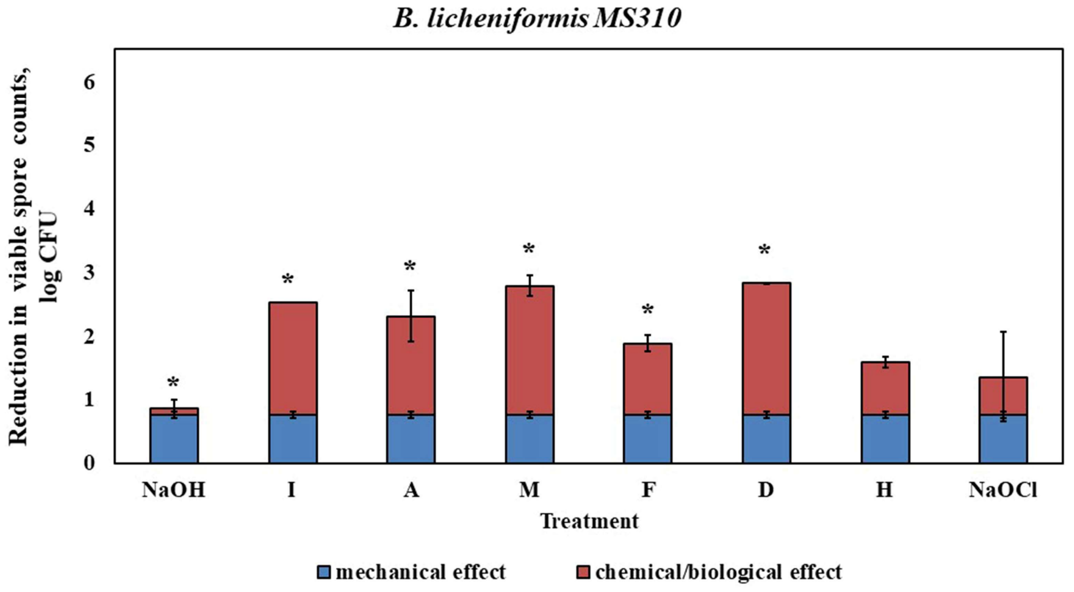

Figure S3. Correlation between mechanical and chemo-biological effect of the examined agents in relation to the removal of the biofilm derived spores in the laboratory CIP system. * – Statistically significant difference ($p < 0.05$) between the reduction in the viable spore counts due to the mechanical effect of liquid circulation and the reduction in the spore counts due to the chemo-biological effect of the examined agents.
